# Factors associated with self-care engagement among oesophageal and gastric cancer survivors: a population-based cross-sectional study

**DOI:** 10.1007/s11136-026-04327-4

**Published:** 2026-06-23

**Authors:** Kenneth Färnqvist, Sara Färnqvist, Asif Johar, Anna Schandl, Pernilla Lagergren

**Affiliations:** 1 Surgical Care Science, Department of Molecular Medicine and Surgery, Karolinska Institutet, Blombäcks väg 23, 4th Floor, 171 77 Stockholm, Sweden; 2 Division of Perioperative- and Intensive Care, Department of Clinical Sciences and Education Södersjukhuset, 118 83 Stockholm, Sweden; 3 Department of Surgery & Cancer, Imperial College London, London, UK

**Keywords:** Stomach neoplasm, Signs and symptoms, Oesophageal neoplasm, Survivors, Self-management, Quality of life

## Abstract

**Background:**

Survivors of oesophageal and gastric cancer frequently experience persistent symptoms and functional limitations that affect their health-related quality of life (HRQL). Although self-care is increasingly recognised as a central component of survivorship care, little is known about what determines self-care engagement or whether engagement is associated with better HRQL. This study examined the sociodemographic and healthcare-related variables associated with self-care engagement and explored its association with global quality of life (QOL) in a nationwide cohort.

**Methods:**

A cross-sectional survey was conducted among individuals diagnosed with oesophageal or gastric cancer in Sweden between 2021 and 2023. Participants completed the EORTC QLQ-C30 and a study-specific questionnaire assessing symptoms and self-care. Self-care engagement was operationalised as the ratio of self-care strategies used to the number of symptoms experienced and categorised into no, moderate, and high. Factors associated with self-care engagement were analysed using multinomial logistic regression, and the association between self-care engagement and global QOL was assessed using linear regression, adjusting for sociodemographic and clinical confounders.

**Results:**

Of the 432 respondents, 422 reported at least one symptom and were included in the self-care analysis. One-third of the participants reported no self-care engagement, whereas two-thirds reported moderate or high engagement. Across all models, university education was the strongest and only consistent variable associated with self-care engagement (high vs. no self-care engagement: odds ratio 2.83, 95% confidence interval 1.48–5.42). Neither assignment of a designated contact nurse nor proximity to the treating hospital was associated with self-care engagement. Participants with high self-care engagement had substantially better global QOL than those with moderate or no self-care engagement, with mean differences exceeding 10 points on a 0–100 scale. Global QOL did not differ between the moderate and no self-care groups.

**Conclusions:**

Self-care engagement varies widely among oesophageal and gastric cancer survivors and appears to be influenced by educational level. High self-care engagement is associated with clinically meaningful improvements in global QOL, whereas no or moderate self-care appears insufficient to confer benefits. These findings suggest an equity gap in survivorship care, and future research should explore the development of structured and accessible interventions to enhance self-care capabilities among patients with lower educational attainment.

**Supplementary Information:**

The online version contains supplementary material available at https://doi.org/10.1007/s11136-026-04327-4.

## Background

The global burden of cancer continues to escalate, with approximately 20 million new cases reported worldwide in 2022 [1]. Oesophageal cancer accounts for one in every 22 cancer-related fatalities globally, ranking seventh in mortality with 445,000 deaths annually and eleventh in incidence with 511,000 new cases annually [1]. Gastric cancer presents an even greater challenge, with nearly one million new cases and approximately 660,000 deaths in 2022, accounting for approximately one in every 15 cancer-related deaths [1]. Although advances in detection and treatment have improved survival rates, survivors often experience long-term sequelae, including fatigue, nutritional issues, and psychological distress [2, 3]. While postoperative follow-up primarily focuses on monitoring recurrence, survivorship care should also address symptom management and health-related quality of life (HRQL) [4].

In the context of cancer survivorship, self-care engagement refers to individuals' activities aimed at managing symptoms, sustaining physical and psychological functioning, and enhancing overall health and well-being outside formal healthcare settings. This conceptualisation is consistent with widely accepted definitions of self-care, including those articulated by the World Health Organisation, which describe self-care as the capacity of individuals to promote and maintain health and to manage illness, with or without the assistance of a healthcare provider [5–7].

Previous research indicates that self-care strategies such as exercise, dietary modifications, and multimodal rehabilitation can enhance cardiorespiratory fitness and HRQL in oesophageal and gastric cancer [8–12]. In this context, self-care usually involves managing persistent postoperative and treatment-related symptoms, including nutritional difficulties, fatigue, and gastrointestinal issues (e.g., reflux or dumping), through behavioural modifications, such as dietary changes, physical activity, pacing, and symptom monitoring [8, 9] However, the findings remain inconsistent across studies, with some interventions demonstrating clinically meaningful benefits, whereas others show limited or no effects [13–16]. This heterogeneity likely reflects differences in patient populations, treatment context, intervention content and intensity, and outcome assessment, as well as small sample sizes and variable adherence. Moreover, while a recent systematic review has synthesised self-care advice for oesophageal cancer survivors [8], no comparable comprehensive synthesis exists for gastric cancer, where the evidence remains fragmented. Consequently, systematic reviews continue to emphasise the lack of clear, consistent, evidence-based guidance on which self-care strategies should be recommended, to whom, and at what point in the care trajectory. Moreover, although considerable attention has been devoted to patients' self-care strategies, understanding of who engages in self-care and the factors influencing this participation is limited. Recognising what determines self-care behaviour is essential to ensure that all survivors, regardless of sociodemographic or clinical characteristics, have equal opportunities to manage their health effectively.

Potential determinants include sociodemographic factors, such as age, sex, and educational level, which can influence health literacy, access to resources, and preferences for self-care [10–12]. Additionally, proximity to the treating hospital may be pertinent, as patients residing farther away often encounter barriers to follow-up care and may consequently rely more on self-care [13, 14]. Furthermore, HRQL itself may function both as a determinant and an outcome of self-care: patients with reduced HRQL may be more inclined to engage in self-care, or less able to do so due to fatigue or psychological distress [6, 15–17].

To the best of our knowledge, no prior research has investigated the determinants of self-care among oesophageal and gastric cancer survivors in a population-based context. Addressing this gap is essential for tailoring survivorship care and developing targeted interventions to enhance patient self-care in this population. Therefore, this study aimed to examine sociodemographic and healthcare-related variables associated with self-care engagement and to explore the association between self-care engagement and global quality of life (QOL) in a nationwide cohort of oesophageal and gastric cancer survivors.

## Research questions

Which sociodemographic factors are associated with self-care engagement?

Is formal assignment of a designated contact nurse associated with self-care engagement?

Does proximity to the treating hospital affect self-care engagement?

Is self-care engagement associated with global quality of life (QOL)?

*Contact nurse, a designated cancer nurse responsible for patient communication, care coordination, and support during survivorship.

## Methods

### Study design

This study followed a prespecified study plan outlined in an internal study protocol and adhered to the same definitions of exposures, outcomes, and confounders. In the present study, we report only quantitative survey data. This nationwide, cross-sectional survey included oesophageal and gastric cancer survivors in Sweden. We followed the Consensus-Based Checklist for Reporting of Survey Studies (CROSS) guidelines [18]. The checklist report is available in the Supplementary Material.

#### Study population and eligibility

Participants were identified through the Swedish National Quality Registry for Oesophageal and Gastric Cancer (NREV), accessible via the Information Network for Cancer Care (INCA). Eligible individuals included adults diagnosed with oesophageal or gastric cancer between 2021 and 2023 who were proficient in Swedish. The tumour site was categorised into mutually exclusive anatomical groups based on registry data. The exclusion criteria were inability to provide informed consent or an in situ tumour only. All eligible patients in the registry were invited to participate, thereby ensuring the study’s population-based representativeness.

#### Survey administration and data collection

Between May 2024 and August 2024, the registry staff distributed invitations that included study information, a paper questionnaire, and a personal QR code that provided a secure link for online completion. Two postal reminders were dispatched to participants who did not respond.

### Clinical and demographic information

Clinical and demographic characteristics were obtained from the NREV. This national platform links clinical cancer data with civil registry information from the Swedish Tax Agency’s Navet Service, enabling the identification of all eligible patients diagnosed between 2021 and 2023.

From the NREV, we retrieved the following variables:

- Patient characteristics included age, sex, and treating hospital.
- Tumour characteristics: histological type and tumour site (ICD-based classification).
- Treatment characteristics: surgical intervention (yes/no, date of operation) and neoadjuvant or adjuvant therapy (type and timing).
- Follow-up status: Vital status and date of last follow-up.

### Questionnaires

Briefly, global QOL was assessed using the EORTC QLQ-C30 [19], complemented by a study-specific questionnaire developed in collaboration with patient partners and healthcare professionals to capture symptom experiences and self-care engagement.

#### The EORTC QLQ-C30 questionnaire

The EORTC QLQ-C30 is a 30-item instrument developed to assess HRQL in individuals with cancer [19]. It includes five multi-item functional scales that assess the physical, role, cognitive, emotional, and social dimensions, as well as a global QOL scale. Symptom burden is evaluated using three multi-item scales addressing fatigue, pain, and nausea/vomiting, as well as six single items that capture common cancer-related issues, such as dyspnoea, insomnia, appetite loss, constipation, diarrhoea, and financial difficulties.

All items except global QOL are rated on a four-point Likert scale (“not at all”, “a little”, “quite a bit”, and “very much”), whereas the global QOL scale comprises two questions scored from 1 (“very poor”) to 7 (“excellent”). For analytical purposes, responses can be dichotomised, with scores of “quite a bit” or “very much” indicating the presence of problems, while lower ratings indicate no or only minor problems. Similarly, a response of 1 or 2 on the global scale is often interpreted as indicating poor overall QOL. All scores were standardised to a 0–100 scale, with higher values in the functional and global domains reflecting better HRQL, whereas higher symptom scores indicated a greater burden.

#### Study-specific questionnaire

A study-specific questionnaire was developed to evaluate symptom experiences and self-care strategies among oesophageal and gastric cancer survivors. This instrument was developed in collaboration with clinical researchers, healthcare professionals, and patient partners to ensure its clinical relevance and patient-centredness. It encompasses the presence and severity of 24 commonly reported symptoms, such as fatigue, nausea, reflux, dumping, and dysphagia, and includes an open-text option for additional symptoms, if any. No formal reliability testing (such as test–retest reliability or internal consistency) was conducted. Nonetheless, construct validity was assessed by evaluating convergent validity with relevant domains of the EORTC QLQ-C30 and by examining known-groups validity across different levels of symptom burden. The results of these analyses are presented in the Supplementary Material.

For each reported symptom, branching logic guided respondents to follow-up questions concerning symptom burden, healthcare contacts, medical diagnosis, interprofessional support, and self-care. Symptom burden was assessed using a five-point Likert scale ranging from 1 (“not at all”) to 5 (“very much”). Healthcare interactions were documented using yes/no questions regarding physician visits and consultations with other professionals. Self-care was evaluated using a yes/no item, followed by free-text descriptions supported by symptom-specific examples to aid recall.

Self-care engagement was assessed using a symptom-specific yes/no item (“Have you treated this symptom with any form of self-care?”). To ensure a shared understanding of the concept, participants were explicitly informed that self-care referred to actions they had undertaken to improve or manage the symptom they reported. This definition was intentionally broad and behaviour-focused, emphasising respondents’ own actions rather than formal healthcare interventions.

In addition to symptom management, the questionnaire collected sociodemographic information, including age, sex, education, and partnership status, as well as self-rated general health on a 5-point Likert scale from “poor” to “excellent”, and proximity to the treating hospital (≤ 50 km vs. > 50 km). Finally, the participants were invited to provide open-ended reflections or advice for other survivors. Convergent validity was assessed using linear regression models that compared study-specific questionnaire items with the corresponding EORTC QLQ-C30 symptom scales, demonstrating good agreement between the instruments. Additionally, both participants and clinicians affirmed the instrument’s face validity. The questionnaire is presented in more detail in the Supplementary Material.

### Patient and public involvement

A structured research partnership group comprising survivors and advocates [20] engaged in the development and refinement of the questionnaire and piloted both paper and web versions. Their contributions ensured that the survey items were relevant and comprehensible.

### Ethical considerations

The study was granted approval by the Swedish regional ethics review authority (2022–05391-01; 2023-04139-02). Participants received written information about the study, and their completion and submission of the questionnaire served as evidence of their informed consent. The data were pseudonymised and securely stored on encrypted servers at the Karolinska Institutet, with access restricted to authorised research personnel.

### Exposures

Sociodemographic and healthcare-related variables were treated as exposures in multinomial logistic regression analyses, with self-care as the outcome. The variables included age (continuous), sex (male/female), educational level (primary, secondary, university), partnership status (partnered/not partnered), time elapsed since diagnosis (continuous), formal assignment of a designated contact nurse (yes/no), and proximity to the treating hospital (≤ 50 km vs. > 50 km).

### Outcomes

- Self-care engagement was operationalised as the ratio of the number of self-care strategies used divided by the total number of reported symptoms. This variable was categorised into three groups based on the 33rd and 66th percentiles: (1) no self-care, (2) ratio < 0.67 (moderate), and (3) ratio ≥ 0.67 (high). The categorisation into three groups was based on tertiles of the distribution to ensure balanced group sizes and stable estimates. Self-care engagement was analysed both as an outcome in the multinomial regression models and as an exposure in the analyses assessing associations with global QOL. Participants without symptoms were excluded from the analyses because self-care engagement was defined as the ratio of self-care strategies to the number of symptoms.
- Global QOL was measured using the EORTC QLQ-C30 global health status scale (0–100, higher = better). In the analyses, global QOL was treated as a continuous outcome, and mean score differences were reported across self-care engagement categories.

### Sample size

In accordance with the descriptive, exploratory survey design, the sample size was determined by precision considerations rather than hypothesis testing, consistent with established recommendations for survey research [23]. Using a standard formula to estimate a proportion with 95% confidence and a margin of error of ± 5 percentage points, it was determined that a minimum of approximately 383 respondents would be sufficient. To enhance coverage, a population-based invitation was extended to all patients diagnosed with and treated for oesophageal or gastric cancer in Sweden over a three-year period. Based on the registry data, approximately 1,000 eligible survivors were anticipated to be identified. With an expected response rate of 60–70%, this approach was projected to yield 600–700 completed questionnaires, corresponding to a margin of error of approximately ± 4 percentage points for proportions near 50% (i.e. the expected proportion of participants with any self-care engagement).

### Statistical analysis

Descriptive statistics were used to summarise demographic and clinical characteristics, symptom burden, global QOL, and self-care engagement. Continuous variables are presented as means with standard deviations (SD) or medians with interquartile ranges (Q1-Q3), and categorical variables are presented as frequencies and percentages.

Self-care engagement, as defined above, was analysed as a categorical variable with three levels (no, moderate, and high self-care).

In the analyses assessing the association between self-care engagement and QOL, we included a predefined set of covariates to reduce confounding. The variables included age (continuous), sex (female/male), educational level (university/secondary/primary), partnership status (partner/ no partner), time since diagnosis (continuous), tumour histology (adenocarcinoma/squamous cell carcinoma), tumour site (oesophagus/gastro-oesophageal junction/stomach), surgical treatment (yes/no), and neoadjuvant or adjuvant therapy (yes/no).

Analyses of self-care engagement were conducted as the outcome in three separate multinomial logistic regression models: Patient characteristics model (model 1) comprised factors associated with self-care engagement, including age, sex, educational level, partnership status, years since diagnosis, and surgical treatment (not adjusted for distance to hospital or contact nurse). The distance-to-hospital model (model 2) included all sociodemographic variables in the patient characteristics model, plus proximity to the treating hospital (≤ 50 km vs. > 50 km). The contact nurse model (model 3) included all sociodemographic variables in the patient characteristics model, plus formal assignment of a designated contact nurse (yes/no). Formal assignment of a designated contact nurse was defined as a nurse formally assigned, according to registry data. In the Swedish cancer care system, patients with oesophageal and gastric cancer are recommended to be assigned a designated contact nurse as part of routine care. However, previous national evaluations and empirical studies have demonstrated variation in how the contact nurse function is implemented, organised, and operationalised across regions, hospitals, and diagnostic pathways [21]. The content, continuity, availability, and organisational prerequisites of contact nurse services may differ between settings. Consequently, this variable should primarily be interpreted as an organisational marker reflecting the implementation of supportive cancer care structures rather than actual use of nursing support or patient–nurse interaction. A stepwise modelling approach was adopted to aid the interpretation of healthcare-related variables. Contact nurse assignment and distance to hospital were analysed in separate models to enable clearer assessment of their associations with self-care engagement. Although both variables may reflect aspects of healthcare accessibility and survivorship care organisation, additional analyses showed no evidence of association or problematic collinearity between them (Pearson’s chi-square p = 0.92; Fisher’s exact p = 0.91; phi/Cramér’s V = 0.005; variance inflation factors approximately 1.1). Results are presented as odds ratios (OR) with 95% confidence intervals (95% CI).

In the analysis of self-care and QOL, the relationship between self-care engagement (categorised by ratio) and QOL (measured using the QLQ-C30 global QOL scale) was assessed using linear regression. QOL was treated as a continuous variable, with results reported as mean score differences (MSD) with 95% CI. The adjustment model mirrored the structure of the multinomial models, incorporating covariates such as age, sex, education level, partnership status, years since diagnosis, and the surgical treatment. All statistical tests were two-sided, and p-values less than 0.05 were considered statistically significant.

Missing data were minimal across variables (generally 1–2% for variables such as education, partnership status, and distance to the hospital), with eight observations (1.9%) excluded due to missing values in either the outcome or explanatory variables for the multinomial models. Given the low level of missing data, no imputation was performed, and analyses were conducted using the complete-case data. The missing data were considered random because the patterns did not vary according to key sociodemographic or clinical characteristics.

To assess potential non-response bias, we compared respondents and non-respondents on key demographic and clinical variables available in the national registry. Sensitivity analyses were not conducted because the missing data were minimal, and the direction and magnitude of the associations remained consistent across all three multinomial models.

Analyses were performed using SAS version 9.4 (SAS Institute Inc., Cary, NC, USA) by an experienced biostatistician (AJ).

## Results

During 2021–2023, 4,144 individuals in Sweden were diagnosed with oesophageal or gastric cancer. Of these, 1585 were alive and residing in Sweden at the time of data collection and were therefore invited to participate in the survey. A total of 489 individuals completed the questionnaire, yielding a 31% response rate. After excluding respondents with in-situ tumours (n = 56) and one case without registry information, 432 participants remained. Of these, 10 reported no symptoms and were therefore excluded from the self-care analyses, resulting in a final analytical sample of 422 individuals for the self-care outcomes.

The mean age of the cohort was 68 years (SD 10.6), ranging from 22 to 86. Approximately 7 out of 10 participants were male. Tumour distribution was heterogeneous: 43% had oesophageal cancer, 31% were diagnosed at the gastroesophageal junction, and 26% had gastric cancer. Most respondents had undergone surgery (71%). Of the participants included in the analyses, 140 (33%) reported no self-care, 150 (36%) moderate self-care, and 132 (31%) high self-care engagement. Participants in the high self-care group also tended to use a broader range of distinct self-care strategies compared to those in the moderate engagement group. A detailed presentation of the demographic and clinical features is provided in Table 1.

**Table 1:** Demographic and clinical characteristics of the study population (n = 422)

Age at biopsy	68 years; SD 10.6
	Number (%)
Histology	
Adenocarcinoma	356 (85)
Squamous cell carcinoma	63(15)
Undifferentiated cancer	2 (< 1)
Gender	
Male	300 (71)
Female	122 (29)
Tumour location	
Cervical oesophagus	1 (< 1)
Upper third of the oesophagus	13 (3)
Middle third of the oesophagus	39 (9)
Lower third of the oesophagus	111 (26)
Oesophagus, unspecified	18 (4)
Cardia Type I	35 (8)
Cardia Type II	53 (13)
Cardia Type III	28 (7)
Unspecified cardia type	15 (4)
Fundus (upper part)	6 (1)
Corpus (middle part)	38 (9)
Antrum (lower part)	27 (6)
Pylorus (lower gastric sphincter)	16 (4)
Lesser curvature, unspecified	2 (< 1)
Malignant tumour of the stomach with overlapping growth	4 (1)
Stomach, unspecified	16 (4)
Time elapsed since the year of diagnosis	1.7 years; SD 0.81
Highest level of education	Number (%)
Primary school	101 (24)
Upper secondary school/high school	149 (36)
University/college, up to 3 years	167 (40)
Partnership status	
Partnered	299 (71)
Not partnered	118 (28)
Surgery	
Yes	298 (72)
No	124 (29)
Proximity to the treating hospital	
≤ 50 km	300 (72)
> 50 km	116 (28)
Assigned contact nurse	
Yes	125 (30)
No	297 (70)
Self-care engagement (ratio of strategies to symptoms)	
No self-care	140 (33)
Moderate self-care	150 (36)
High self-care	132 (31)
	Median (Q1- Q3)
Number of self-care strategies used	
Whole cohort	2 (0–6)
No self-care	0 (0)
Moderate self-care	3 (1–4)
High self-care	7.5 (5–12)
Number of symptoms	
Whole cohort	8 (5–11)
No self-care	7 (4–10)
Moderate self-care	9 (6–13)
High self-care	7 (4–11)
	Mean score (SD)
Unadjusted Global QOL	
Whole cohort	66.51 (22.25)
No self-care	63.13 (23.46)
Moderate self-care	63.91 (22.45)
High self-care	73.00 (19.3)

SD, standard deviation; QOL, quality of life; Q1–Q3, interquartile range (25th–75th percentile)

Comparison with registry-based data indicated that respondents and non-respondents were broadly similar in terms of demographic and clinical characteristics. A minor difference was noted in the treatment intent, with palliative therapy being slightly less common among respondents (see Supplementary Material).

### Symptom burden and number of self-care strategies across engagement groups

Participants with no self-care reported a median of 7 symptoms (Q1–Q3:4–10) and, by definition, did not use any strategies. Those in the moderate self-care group reported a higher symptom burden (median 9 symptoms, Q1–Q3:6–13) and used a median of three strategies (Q1–Q3:1–4). Participants in the high self-care group reported a symptom burden comparable to the no self-care group (median 7 symptoms, Q1–Q3:4–11), but used markedly more strategies (median 7.5, Q1–Q3:5–12).

Unadjusted global QOL scores mirrored these patterns, with the high self-care group reporting the highest global QOL (mean 73.0, SD 19.3), compared with substantially lower scores among the moderate (mean 63.9, SD 22.5) and no self-care groups (mean 63.1, SD 23.5).

### Patient characteristics model: factors associated with self-care engagement

Participants with a university education were significantly more likely to engage in self-care. Compared with those with only primary education, university-educated individuals had higher odds of high self-care (OR 2.83, 95% CI 1.48–5.42) and moderate self-care (OR 2.44, 95% CI 1.29–4.61) engagement compared to no self-care. Secondary education was not associated with self-care engagement (OR range: 1.22–1.38). The full model is presented in Table 2.

**Table 2:** Associations between sociodemographic and healthcare-related characteristics and levels of self-care engagement among oesophageal and gastric cancer survivors

Variable	Comparison	Model 1 high vs no self-care OR (95% CI)	Model 1 moderate vs no self-care OR (95% CI)	Model 2 high vs no self-care OR (95% CI)	Model 2 moderate vs no self-care OR (95% CI)	Model 3 high vs no self-care OR (95% CI)	Model 3 moderate vs No self-care OR (95% CI)
Age (years)	Per year ↑	0.995 (0.971–1.020)	0.988 (0.965–1.012)	0.99 (0.97–1.02)	0.99 (0.96–1.01)	0.99 (0.97–1.02)	0.99 (0.96–1.01)
Sex	Female vs male	1.48 (0.86–2.57)	0.87 (0.50–1.52)	1.49 (0.86–2.58)	0.88 (0.50–1.54)	1.49 (0.86–2.57)	0.88 (0.50–1.54)
Education							
	University vs primary	2.83 (1.48–5.42)	2.44 (1.29–4.61)	2.81 (1.47–5.38)	2.36 (1.25–4.47)	2.83 (1.48–5.43)	2.45 (1.30–4.62)
	Secondary vs primary	1.22 (0.63–2.37)	1.38 (0.74–2.56)	1.22 (0.63–2.37)	1.39 (0.75–2.59)	1.22 (0.63–2.37)	1.37 (0.74–2.54)
Partnership status	Partnered vs not partnered	1.00 (0.58–1.72)	1.33 (0.77–2.29)	1.01 (0.58–1.73)	1.36 (0.79–2.35)	1.00 (0.58–1.72)	1.34 (0.78–2.31)
Years since diagnosis	per year ↑	1.01 (0.74–1.37)	1.03 (0.77–1.38)	1.01 (0.74–1.37)	1.03 (0.76–1.38)	1.00 (0.72–1.38)	1.01 (0.74–1.37)
Surgery received	Yes vs no	0.73 (0.42–1.26)	1.23 (0.71–2.13)	0.73 (0.43–1.26)	1.25 (0.72–2.17)	0.73 (0.42–1.26)	1.21 (0.70–2.11)
Distance to hospital	> 50 km vs ≤ 50 km	–	–	0.91 (0.53–1.57)	0.64 (0.38–1.10)	–	–
Assigned contact nurse	Yes vs no	–	–	–	–	0.97 (0.55–1.71)	0.87 0.50–1.51)

Model 1 adjusted for age, sex, education, partnership status, years since diagnosis, and surgery. Model 2 additionally adjusted for distance to the treating hospital. Model 3 additionally adjusted for an assigned contact nurse. The reference category for the outcome was no self-care engagement. Odds ratios (OR) with 95% CI are presented. Eight observations were excluded due to missing responses in the dependent or independent variables. OR = odds ratio; CI = confidence interval

### Distance-to-hospital model: factors associated with self-care engagement

University education was associated with higher self-care engagement, with increased odds of both high (OR 2.81, 95% CI 1.47–5.38) and moderate self-care (OR 2.36, 95% CI 1.25–4.47) relative to no self-care. Secondary education showed no clear association (OR range: 1.22–1.39). Distance to the treating hospital was not associated with self-care engagement (high self-care: OR 0.91, 95% CI 0.53–1.57; moderate self-care: OR 0.64, 95% CI 0.38–1.10). The full model is presented in Table 2.

### Contact nurse model: factors associated with self-care engagement

University education remained the only variable consistently associated with self-care engagement. Compared with primary education, university-educated participants had higher odds of high (OR 2.83, 95% CI 1.48–5.43) and moderate self-care (OR 2.45, 95% CI 1.30–4.62) relative to no self-care. Secondary education did not differ significantly (OR range: 1.22–1.37).

Formal assignment of a designated contact nurse was not associated with high self-care engagement (OR 0.97, 95% CI 0.58–1.62) and moderate self-care (OR 0.87, 95% CI 0.53–1.42). The full model is presented in Table 2.

### Association between self-care engagement and global QOL

Higher levels of self-care engagement were associated with higher global QOL. The adjusted mean scores increased progressively across the three self-care groups, with the highest values observed among participants with high self-care (71.84), followed by those with moderate self-care (61.65), and the lowest among individuals who reported no self-care (61.08). When comparing estimated mean differences, the high self-care group scored, on average, 10.19 points higher than the moderate self-care group (95% CI 4.99–15.38) and 10.75 points higher than participants practising no self-care (95% CI 5.42–16.09). The difference between the moderate and no self-care groups was minimal and not statistically significant (0.57 points, 95% CI –4.58 to 5.71). These results demonstrate that increased engagement in self-care strategies is correlated with higher global QOL scores.

## Discussion

In this nationwide cohort study of oesophageal or gastric cancer survivors, we observed significant variations in individuals' engagement with self-care strategies following treatment. One-third of participants reported no engagement in self-care activities, while a similar proportion engaged at varying levels of self-management. Across all analytical models, higher education emerged as the most robust and consistent predictor of self-care. Neither formal assignment of a designated contact nurse nor geographic proximity to the treating hospital was associated with self-care activities. Notably, participants in the high self-care group reported significantly better global QOL than those in the moderate or no self-care groups, with an average difference exceeding 10 points, which is typically considered clinically significant. In contrast, the difference between the moderate and no self-care groups was minimal, suggesting that lower levels of engagement may not be sufficient to meaningfully influence well-being or that a higher symptom burden may be associated with reduced capacity to engage consistently with self-care.

### Strengths and limitations

This study had several notable strengths. The use of population-based sampling enhances the representativeness and generalisability of the findings to Swedish survivors of oesophageal and gastric cancers. The large national cohort further facilitated the robust modelling of sociodemographic and clinical variables. However, this study has some limitations. The cross-sectional design precludes causal inference, preventing the determination of whether self-care improves global QOL or whether individuals with better global QOL are more inclined to engage in it. Self-reported measures introduce the risk of recall and reporting bias, particularly for subtle or intermittent behaviours. The response rate was modest (~ 31%), although a comparison of non-responders with responders suggested broadly similar demographic and clinical characteristics. Nonetheless, respondents may represent a more health-engaged population, potentially underestimating the true inequities in self-care. This may also indicate a degree of survival-related selection. Although a comparison with non-responders suggested broadly similar clinical characteristics, the findings may still be less relevant to patients with more advanced diseases or poorer prognoses.

### Interpretation and conceptual relevance

The association between self-care engagement and enhanced global QOL may reflect a bidirectional relationship between capabilities and self-control. Individuals in better health may experience increased motivation, physical ability, and mental readiness to manage their symptoms actively. Conversely, self-care may also enhance global QOL through mechanisms such as reducing symptom burden, increasing perceived control, and improving self-efficacy [17]. More broadly, the findings of this study can be interpreted within established behavioural models of self-management, which emphasise the role of both cognitive and contextual factors in shaping self-care behaviours. This aligns with research on habit formation [22] and the Common-Sense Model of illness representation, in which both beliefs and routines influence coping behaviours [23, 24].

It should also be acknowledged that educational attainment has consistently been associated with HRQL across cancer populations, independent of specific self-care behaviours. Studies have shown that individuals with higher education generally report better global QOL, physical functioning, and fewer long-term symptoms, likely reflecting differences in health literacy, coping resources, and broader social determinants of health [25–27]. Consequently, part of the observed association between high self-care engagement and improved global QOL may reflect an underlying socioeconomic gradient rather than a direct effect of self-care alone [28].

Against this background, the absence of a distinct difference between moderate and no self-care suggests that sporadic or irregular strategies may be insufficient; rather, consistent, intentional, and diverse efforts to manage symptoms may be necessary to achieve significant improvements in well-being. The descriptive data further support this perspective. Participants in the moderate self-care group reported the highest symptom levels; however, their global QOL was comparable to that of participants who did not engage in self-care. In contrast, the high self-care engagement group experienced a symptom burden similar to that of the no self-care group but reported a substantially higher global QOL. Together, these patterns suggest that while self-care engagement may be related to symptom burden, only more extensive engagement appears associated with higher QOL, although the direction of this relationship cannot be determined in this cross-sectional design. Participants with higher self-care engagement also tended to use a broader range of self-care strategies, which partly reflects the greater number of strategies used overall, but may also indicate a broader behavioural repertoire in symptom management.

### Self-care in the context of previous research

Research on self-care among upper gastrointestinal cancer survivors remains limited, and a recent systematic review showed that much of the available advice is based on experiential knowledge rather than formal evidence [8]. Our quantitative findings support this by demonstrating that self-care engagement is not uniformly distributed among survivors, with significantly higher levels observed among those with a university education than among those without. This suggests that in the absence of formalised and accessible self-care guidance, survivors with greater health literacy and more informational resources may be better equipped to engage in effective self-care, potentially exacerbating socioeconomic disparities [29]. Research on other cancer cohorts and chronic diseases similarly shows that educational attainment, health literacy, and access to informational resources significantly influence self-management behaviours [30–32]. Our study extends these findings to oesophageal and gastric cancers, revealing that disparities persist even within a publicly funded healthcare system offering universal access. Neither proximity to the treating hospital nor formal assignment of a designated contact nurse was associated with self-care engagement, suggesting that structural access alone does not determine self-management. We explored whether this association differed by educational level, but found no evidence of interaction. However, the contact nurse variable reflects formal assignment rather than actual use of nursing support and may partly capture broader organisational and healthcare accessibility factors, including regional variation in the implementation of survivorship care. Although no statistical association or problematic collinearity was observed between contact nurse assignment and geographical distance, both variables may still reflect organisational aspects of survivorship care delivery rather than direct measures of care use. The present study introduces a quantitative dimension that has, for the most part, been absent from previous literature. While the systematic review described above [8] categorised self-care strategies into domains such as dietary adaptation, symptom monitoring, physical activity, and pacing, it did not provide evidence regarding the extent to which survivors actually employ these strategies or whether increased engagement leads to improved outcomes. By defining self-care as a ratio of strategies to symptom burden and associating high engagement with clinically significant improvements in global QOL, our findings support the review’s [8] qualitative insight that effective self-care requires deliberate, consistent, and effortful practice. The lack of a meaningful difference between moderate and no self-care underscores that sporadic or occasional strategies are unlikely to improve patients’ well-being.

### Clinical and research implications

The robust association between university education and high levels of self-care engagement highlights a potential disparity in survivorship outcomes [12]. Interventions designed to support patients with lower educational attainment, such as personalised coaching, simplified symptom guides, structured follow-up, and digital self-monitoring tools, could mitigate this gap [9, 32–34]. Given the current paucity of quantitative evidence on self-care in upper gastrointestinal cancer, future research should focus on developing and evaluating structured, evidence-based interventions. Such research is important to ascertain which self-care strategies effectively enhance patient outcomes and ensure equitable support across diverse survivor groups. Longitudinal studies are particularly essential; establishing temporality would elucidate whether self-care drives recovery or whether improved recovery facilitates self-care. A randomised or stepped-wedge design could evaluate whether actively enhancing self-care capacity results in measurable improvements in HRQL.

## Conclusion

In this national cohort of oesophageal and gastric cancer survivors, the degree of self-care engagement exhibited considerable variation, with individuals engaging in higher levels of self-care reporting significantly improved global QOL. University education emerged as the most robust variable associated with self-care, highlighting a socioeconomic gradient with potential implications for equity in survivorship outcomes. Although causality cannot be definitively established, these findings suggest that effective self-care may be a viable target for intervention. Future research should investigate whether structured self-care support can mitigate disparities and enhance HRQL following treatment for oesophageal or gastric cancer.

## Supplementary Information

Below is the link to the electronic supplementary material.

Supplementary Material 1

## Data Availability

The datasets examined in this study are not publicly accessible due to ethical constraints, but may be made available from the corresponding author upon reasonable request and subject to applicable approvals.
