# Supplementary material for: Factors associated with self-care engagement among oesophageal and gastric cancer survivors: a population-based cross-sectional study
Source: Qual Life Res. 2026 Jun 23;35(8):215. doi: 10.1007/s11136-026-04327-4 (PMC13291034; doi:10.1007/s11136-026-04327-4)
Supplement: Supplementary file 1 — Supplementary Material 1 [file 11136_2026_4327_MOESM1_ESM.docx]

**Study specific questionnaire**

A study-specific questionnaire was created to examine common post-treatment symptoms among oesophageal and gastric cancer survivors, with an emphasis on self-care practices. The questionnaire was developed in collaboration with senior clinical researchers, specialists in oesophageal and gastric cancers, healthcare professionals, and patient partners from a research partnership group. Formal reliability testing (such as test–retest reliability or internal consistency) was not conducted. However, construct validity was assessed through convergent validity with relevant domains of the EORTC QLQ-C30 and by examining known-groups validity across various levels of symptom burden. Analyses supported the construct validity of the questionnaire, with study-specific symptom items showing expected associations with the corresponding EORTC QLQ-C30 domains and clear gradients in HRQL across symptom-burden levels. See the table below for the tested items.

| **Validation type** | **Study-specific variable** | **Comparator (EORTC QLQ-C30)** | **N** | **Mean (No burden)** | **Mean (High burden)** | **Difference** | **p-value** |
| --- | --- | --- | --- | --- | --- | --- | --- |
| Convergent validity | Fatigue item | Fatigue (FA) | 319 | 32.8 | 57.6 | +24.7 | <0.001 |
| Convergent validity | Nausea/vomiting item | Nausea/Vomiting (NV) | 166 | 19.2 | 35.3 | +16.1 | <0.001 |
| Convergent validity | Insomnia item | Insomnia (SL) | 143 | 41.2 | 72.1 | +30.9 | <0.001 |
| Convergent validity | Diarrhoea item | Diarrhoea (DI) | 211 | 21.5 | 46.8 | +25.3 | <0.001 |
| Convergent validity | Constipation item | Constipation (CO) | 99 | 26.9 | 47.4 | +20.5 | 0.0004 |
| Convergent validity | Pain item | Pain (PA) | 118 | 31.9 | 51.9 | +20.0 | <0.001 |
| Convergent validity | Dyspnoea item | Dyspnoea (DY) | 40 | 55.1 | 78.4 | +23.4 | 0.0056 |
| Known-groups validity | Symptom burden groups (0 / 1 / ≥2) | QLQ-C30 summary score | 411 | 91.2 / 84.1 / 70.0 | – | ↓ with burden | <0.001 |
| Known-groups validity (correlation) | Total symptom burden | QLQ-C30 summary score | 411 | – | – | r = -0.68 | <0.001 |

Construct validity of the study-specific questionnaire was evaluated through convergent validity with the relevant EORTC QLQ-C30 domains and known-groups validity across different symptom burden levels. Higher scores on symptom scales indicate worse symptoms, while higher scores on the summary score reflect better overall health.

The questionnaire employed branching logic, meaning that the structure of the survey was dynamically adapted based on the participants' responses. This ensured that only relevant follow-up questions were displayed for each participant. When logging into the web-based survey interface, participants first encountered the question: “Have you experienced any of the following symptoms during the past six months or earlier?”A list of 24 frequently reported symptoms was followed (e.g. fatigue, nausea, reflux, dumping, and dysphagia), along with an open text box for respondents to report additional symptoms not included in the predefined list. A series of follow-up questions were then triggered for each reported symptom (yes/no).The follow-up items included the following:

- **Symptom burden:** “How much has this symptom bothered you in the past six months?” (rated on a 5-point Likert scale from 1 = “not at all” to 5 = “very much”)
- **Healthcare contact:**
  - “Have you seen a physician specifically for this symptom?” (yes/no)
  - “Have you seen a physician for this symptom during the past 3 months?” (yes/no)
- **Diagnosis:**
  - “Has this symptom been medically diagnosed?” (yes/no)
  - “What was the diagnosis?” (free-text response)
- **Interprofessional support:** “Have you consulted any of the following professionals about this symptom in the past six months?” (checkbox list including, e.g., dietician, physiotherapist, nurse)
- **Self-care:** “Have you treated this symptom with any form of self-care?” (yes/no)

If the participant answered “yes” to the self-care question, a follow-up prompt appeared: “We are especially interested in what actions you have taken to manage this symptom. Click here to see examples.” The examples were tailored to the specific symptoms reported, ensuring that the suggestions were relevant and plausible to the user. The participants were then invited to provide free-text responses describing the self-care strategies they used. Finally, they were asked to rate how effective they believed their self-care had been: “How effective do you think your self-care was for this symptom?” (rated on a 5-point scale from 1 = “not at all” to 5 = “very well”).

Along with symptom- and care-related questions, the questionnaire also collected sociodemographic information, including age, sex (male, female, prefer not to say), highest educational level (primary school, secondary school, university ≥3 years), marital status (e.g. married/partnered, single, divorced, widowed), and self-rated general health (rated on a 5-point Likert scale from 1 = “poor” to 5 = “excellent”). Participants were also asked to indicate the distance to their treating hospital (e.g. <10 km, <30 km, <50 km, >, and >50 km). Finally, an open-ended question allowed respondents to share advice or reflections that they believed might be helpful to others with the same diagnosis.

**Non-responder analysis. Comparison of demographic and clinical characteristics between survey responders and non-responders.**

Data derived from national cancer registry records. Minor differences were observed in the proportion receiving palliative treatment (lower among responders), while other characteristics were broadly similar between the groups.

| **Characteristic** | **Non-responders (n=726)** | **Responders (n=433)** | **Comment** |
| --- | --- | --- | --- |
| Sex (male, %) | 65.6% | 70.4% | Similar distribution |
| Median age at diagnosis (years) | 72.0 | 71.0 | Comparable |
| T stage (T2 or higher, %) | ~69% | ~75% | Comparable |
| N stage (N1–3, %) | 49.5% | 53.1% | Comparable |
| M stage (M1, %) | 20.7% | 18.2% | Comparable |
| Operation performed (%) | 56.6% | 68.8% | Slightly higher in responders |
| Neoadjuvant therapy (%) | 34.7% | 47.6% | Higher in responders |
| Palliative treatment (%) | 14.9% | 11.1% | Slightly lower in responders |
| Definitive chemoradiotherapy (%) | 10.3% | 10.4% | No difference |

**Classification framework for self-care strategies reported by oesophageal and gastric cancer survivors**

| **Category** | **Description** |
| --- | --- |
| **Daily activities** | Gardening; grocery shopping; clothes shopping; chopping wood; clearing brush; working; standing up; trying to remain active; farm work; walking the dog; painting the house; varnishing the deck; physical labour. |
| **Dietary adjustments** | Avoiding vegetables with skins or seeds; switching to lactose-free products; mixed diet; “eating correctly”; eating blueberries every morning; smaller portions; binding foods; lactose-free or low-fat foods; healthier food; gluten-free and lactose-free diet; chewing more thoroughly and learning which foods pass; cutting food into smaller pieces; increasing energy intake; honey; liquid diet; meal replacements with nutritional drinks; fasting; extra toppings; adding butter/cream to food; recording diet in an app; bulk agents; eating crispbread; drinking plenty of fluids; chewing gum; honey water; oregano oil; limiting alcohol; drinking through a straw; dietary supplements. |
| **Medication** | Use of Dimor (loperamide) occasionally or regularly; Imodium; Stelara injections every 12 weeks; sleeping pills; melatonin; nutraceuticals; probiotics; drops; Creon; Inolaxol; antidepressants (citalopram, venlafaxine); expectorants; Primperan; topical creams (urea, carbamide); Movicol; laxatives (Cilaxoral, Microlax); Voltaren gel; morphine; Ventoline inhaler; oral care agents (Exerodent, Xylimelts, Andolex); topical salves (Idomin, Nystimex); Ondansetron; Betapred; CBD oil; Temesta; proton-pump inhibitors (lansoprazole, etc.); over-the-counter remedies (Rennie, Gaviscon, Samarin); cough suppressants; herbal remedies; mouthwash. |
| **Monitoring and pacing** | Keeping a diary; listening to bodily signals; spouse keeping notes; using an Apple Watch to monitor activity; distributing energy throughout the day; walking to distract from symptoms; trying to reduce stress; taking notes on symptoms; practising careful toileting (not straining); adapting medications; adjusting pace. |
| **Motivation and attitude** | “Keeping up the fight”; trying to live as before and maintain good spirits; staying active as much as possible; exercising harder; positive outlook; being kind to oneself; accepting the situation; distraction from worries; thinking of other things; learning to live with it; learning to know one’s body. |
| **No intervention / letting time pass** | Waiting for symptoms to resolve spontaneously; “it got better by itself”; “no particular measure”; improvement over time without intervention. |
| **Physical exercise** | Walking; strength training; gym workouts; stationary cycling; yoga; aikido; swimming; tennis; padel; bowling; pétanque; home exercise; vibration plate; pool exercise; stretching; golf; spinning; running; group training; personal training; morning exercise; general physical activity. |
| **Rest and recovery** | Rest; daytime naps (approximately one hour daily); midday recovery; additional sleep; resting after meals; relaxation. |
| **Sleep management** | Regular sleep routines; sleeping partly upright in a chair or recliner; going to bed at set times; testing different positions and pillow heights; listening to audiobooks to fall asleep; acceptance of sleep difficulties; maximising daytime activity to support sleep; relaxation exercises; cool and dark bedroom; using adjustable bed; segmented sleep; reading until sleepy. |
| **Social and recreational** | Spending time with friends; socialising; walking with acquaintances; handicrafts; reading; meditation/mindfulness; massage; conversations with spouse; going out during the day; meaningful activities; enjoying wine or whiskey; forest walks; cooking; massage device; moving without exertion; phone calls with friends; self-rewarding with gifts; spa visits. |
| **Therapies and treatment** | Physiotherapy; red-light therapy; treatment for benign paroxysmal positional vertigo; conversations with nurse coordinator; posture vest; use of diapers; smell training; orthotic shoe inserts; wool socks at night; compression stockings; heating pads; medical consultations; breathing exercises; scar massage; oral hygiene; balance training; swallowing exercises; reducing snus use; PEP breathing training; huffing; saline steam inhalation; walking with rollator or crutches; elevating feet; psychosocial counselling; osteopathy; assisted coughing/clearing; domestic help. |
| **Non-interpretable response** | No answer; “see above”; “too tired to answer”; irrelevant responses (“the chemo caused this”, “same as before”, “nothing more”); expressions of frustration with the question. |

Free-text responses describing self-initiated symptom management strategies were categorised into 12 conceptual domains. Categories 1–11 represent interpretable and actionable self-care approaches (e.g., dietary modification, pacing, physical exercise, rest, social engagement). Category 12 contains responses that could not be meaningfully interpreted as self-care behaviours, or irrelevant entrie

|  |  |  |  |
| --- | --- | --- | --- |
| **CROSS Reporting Checklist** | | |  |
| **Section/topic** | **Item** | **Item description** | **Reported on page #** |
| **Title and abstract** | | |  |
| Title and abstract | 1a | State the word “survey” along with a commonly used term in title or abstract to introduce the study’s design. | 2 |
|  | 1b | Provide an informative summary in the abstract, covering background, objectives, methods, findings/results, interpretation/discussion, and conclusions. | 2 |
| **Introduction** | | |  |
| Background | 2 | Provide a background about the rationale of study, what has been previously done, and why this survey is needed. | 3 |
| Purpose/aim | 3 | Identify specific purposes, aims, goals, or objectives of the study. | 3 |
| **Methods** | | |  |
| Study design | 4 | Specify the study design in the methods section with a commonly used term (e.g., cross-sectional or longitudinal). | 4 |
|  | 5a | Describe the questionnaire (e.g., number of sections, number of questions, number and names of instruments used). | 5-6 + supplement |
| Data collection methods | 5b | Describe all questionnaire instruments that were used in the survey to measure particular concepts. Report target population, reported validity and reliability information, scoring/classification procedure, and reference links (if any). | 5-6 + supplement |
|  | 5c | Provide information on pretesting of the questionnaire, if performed (in the article or in an online supplement). Report the method of pretesting, number of times questionnaire was pre-tested, number and demographics of participants used for pretesting, and the level of similarity of demographics between pre-testing participants and sample population. | 6 |
|  | 5d | Questionnaire if possible, should be fully provided (in the article, or as appendices or as an online supplement). | Supplement |
| Sample characteristics | 6a | Describe the study population (i.e., background, locations, eligibility criteria for participant inclusion in survey, exclusion criteria). | 4 |
|  | 6b | Describe the sampling techniques used (e.g., single stage or multistage sampling, simple random sampling, stratified sampling, cluster sampling, convenience sampling). Specify the locations of sample participants whenever clustered sampling was applied. | 4 |
|  | 6c | Provide information on sample size, along with details of sample size calculation. | 7 |
|  | 6d | Describe how representative the sample is of the study population (or target population if possible), particularly for population-based surveys. | 4 |
| Survey  administration | 7a | Provide information on modes of questionnaire administration, including the type and number of contacts, the location where the survey was conducted (e.g., outpatient room or by use of online tools, such as SurveyMonkey). | 4 |
|  | 7b | Provide information of survey’s time frame, such as periods of recruitment, exposure, and follow-up days. | 4 |
|  | 7c | Provide information on the entry process:  –>For non-web-based surveys, provide approaches to minimize human error in data entry.  –>For web-based surveys, provide approaches to prevent “multiple participation” of participants. | 4 |
| Study preparation | 8 | Describe any preparation process before conducting the survey (e.g., interviewers’ training process, advertising the survey). | 6 |
| Ethical considerations | 9a | Provide information on ethical approval for the survey if obtained, including informed consent, institutional review board [IRB] approval, Helsinki declaration, and good clinical practice [GCP] declaration (as appropriate). | 6 |
|  | 9b | Provide information about survey anonymity and confidentiality and describe what mechanisms were used to protect unauthorized access. | 4 & 6 |
| Statistical  analysis | 10a | Describe statistical methods and analytical approach. Report the statistical software that was used for data analysis. | 7-8 |
|  | 10b | Report any modification of variables used in the analysis, along with reference (if available). | 7-8 |
|  | 10c | Report details about how missing data was handled. Include rate of missing items, missing data mechanism (i.e., missing completely at random [MCAR], missing at random [MAR] or missing not at random [MNAR]) and methods used to deal with missing data (e.g., multiple imputation). | 8 |
|  | 10d | State how non-response error was addressed. | 9 |
|  | 10e | For longitudinal surveys, state how loss to follow-up was addressed. | N/A |
|  | 10f | Indicate whether any methods such as weighting of items or propensity scores have been used to adjust for non-representativeness of the sample. | N/A |
|  | 10g | Describe any sensitivity analysis conducted. | 8 |
| **Results** | | |  |
| Respondent characteristics | 11a | Report numbers of individuals at each stage of the study. Consider using a flow diagram, if possible. | 8-9 |
|  | 11b | Provide reasons for non-participation at each stage, if possible. | 8-9 |
|  | 11c | Report response rate, present the definition of response rate or the formula used to calculate response rate. | 8 |
|  | 11d | Provide information to define how unique visitors are determined. Report number of unique visitors along with relevant proportions (e.g., view proportion, participation proportion, completion proportion). | 4 & 6 |
| Descriptive  results | 12 | Provide characteristics of study participants, as well as information on potential confounders and assessed outcomes. | 9 + table 1 |
| Main findings | 13a | Give unadjusted estimates and, if applicable, confounder-adjusted estimates along with 95% confidence intervals and p-values. | 9-10 + figure 1-3 |
|  | 13b | For multivariable analysis, provide information on the model building process, model fit statistics, and model assumptions (as appropriate). | 7-10 |
|  | 13c | Provide details about any sensitivity analysis performed. If there are considerable amount of missing data, report sensitivity analyses comparing the results of complete cases with that of the imputed dataset (if possible). | 8 |
| **Discussion** | | |  |
| Limitations | 14 | Discuss the limitations of the study, considering sources of potential biases and imprecisions, such as non-representativeness of sample, study design, important uncontrolled confounders. | 11-12 |
| Interpretations | 15 | Give a cautious overall interpretation of results, based on potential biases and imprecisions and suggest areas for future research. | 12 |
| Generalizability | 16 | Discuss the external validity of the results. | 11-12 |
| **Other sections** | | |  |
| Role of funding source | 17 | State whether any funding organization has had any roles in the survey’s design, implementation, and analysis. | 13 |
| Conflict of interest | 18 | Declare any potential conflict of interest. | 14 |
| Acknowledgements | 19 | Provide names of organizations/persons that are acknowledged along with their contribution to the research. | 13 |
